# Reply to Janssens and Mech: The possibility of natural wolf dispersal to Stora Karlsö

**DOI:** 10.1073/pnas.2608649123

**Published:** 2026-06-01

**Authors:** Linus Girdland-Flink, Anders Bergström, Jan Storå, Erik Ersmark, Jan Apel, Maja Krzewińska, Love Dalén, Anders Götherström, Pontus Skoglund

**Affiliations:** ^a^ Department of Archaeology, University of Aberdeen, Aberdeen AB24 3UF, United Kingdom; ^b^ School of Biological and Environmental Sciences, Liverpool John Moores University, Liverpool L3 3AF, United Kingdom; ^c^ School of Biological Sciences, University of East Anglia, Norwich NR4 7TJ, United Kingdom; ^d^ Ancient Genomics Laboratory, The Francis Crick Institute, London NW1 1AT, United Kingdom; ^e^ Department of Archaeology and Classical Studies, Stockholm University, Stockholm SE-106 91, Sweden; ^f^ Centre for Palaeogenetics, Stockholm SE-106 91, Sweden; ^g^ Department of Zoology, Stockholm University, Stockholm SE-106 91, Sweden; ^h^ Department of Bioinformatics and Genetics, Swedish Museum of Natural History, Stockholm SE-104 05, Sweden

In their letter about our article describing the discovery of prehistoric wolves on the small island of Stora Karlsö (1), Janssens and Mech argue that natural dispersal is a more likely explanation than human agency (2). After reviewing their arguments, we still favor human agency, but maintain, as we emphasized in the article, that natural dispersal cannot be excluded.

1. Janssens and Mech correctly point out that the distance to Wrangel Island is ~140 km, not 70 km as stated in Linnell et al. (3), and used to define the “extreme edge” that we paraphrased. But unlike the East Siberian Sea around Wrangel Island, the southern Baltic Sea only rarely freezes over to create sustained ice corridors suitable for crossing (4). Moreover, conditions during the Holocene Thermal Maximum (8,000 to 4,800 years BP), close to when the oldest SF wolf lived (~4.7 kya), were likely even less favorable due to higher temperatures and salinity levels (5, 6). Finally, the assertion of wolf ice crossings over the Baltic Sea of >150 km is not supported by the cited paper. The longest stretch of uninterrupted ice on that 150 km route is only ~40 km through the Åland archipelago (3). And importantly, wolves crossing the Baltic Sea remain a hypothesis, not an observation.
2. Their second point misinterprets the isotope data to suggest that the marine diet signal in both wolves derived mainly from seals. The isotope values for G.7 (figure 2*C* in ref. 1) are instead consistent with a high proportion of lower tropic level marine protein such as fish. While coastal Alaskan wolves are known to hunt fish in shallow rivers and estuaries (7, 8), the lack of such shallow-water niches on or around Stora Karlsö would make regular piscivorous behavior challenging.
3. The regression weight estimate is not contradicting but rather validating our conclusions. They estimate G.11’s weight at 32.3 kg, at the lower end of the range for wild wolves (31–46 kg). This matches our statement that the G.11 humerus sits at the lower end of the size range of Pleistocene and Holocene wolves (figure 1*B* in ref. 1).
4. The critique misinterprets “rarity” as absolute scarcity rather than frequency relative to more ubiquitous taxa. We cite literature to exemplify a diverse range of possible human–wolf interactions in the archaeological record (9).
5. We did not claim that humans cared for the G.11 wolf nor that it was lame, only that the pathology likely reduced its mobility.

Finally, the strong assertions drawn from the radiocarbon age gap between the two wolves ignore the complex chronology of Scandinavia. A Middle Neolithic date (G.7) allows in principle for cultural affiliation to Pitted Ware Culture, Funnelbeaker, or even Battle Axe culture (10). This opens the possibility for some degree of genetic and cultural affinity with later Bronze Age societies (G.11) but addressing this would require comprehensive coanalysis of the extensive archaeological assemblage and falls beyond the scope of the present study.
